# Zinc Inhibits Expression of Androgen Receptor to Suppress Growth of Prostate Cancer Cells

**DOI:** 10.3390/ijms19103062

**Published:** 2018-10-08

**Authors:** Phuong Kim To, Manh-Hung Do, Young-Suk Cho, Se-Young Kwon, Min Soo Kim, Chaeyong Jung

**Affiliations:** 1 Department of Anatomy, Chonnam National University Medical School, Gwangju 61469, Korea; tkphuong2609@gmail.com (P.K.T.); manhhung.cnsh@gmail.com (M.-H.D.); tonytoy@hanmail.net (Y.-S.C.); candy8900@naver.com (S.-Y.K.); 2 Department of Statistics, College of Natural Sciences, Chonnam National University, Gwangju 61186, Korea; kimms@jnu.ac.kr

**Keywords:** zinc, prostate cancer, androgen receptor

## Abstract

The prostate gland contains a high level of intracellular zinc, which is dramatically diminished during prostate cancer (PCa) development. Owing to the unclear role of zinc in this process, therapeutic applications using zinc are limited. This study aimed to clarify the role of zinc and its underlying mechanism in the growth of PCa. ZnCl_2_ suppressed the proliferation of androgen receptor (AR)-retaining PCa cells, whereas it did not affect AR-deficient PCa cells. In LNCaP and TRAMP-C2 cells, zinc downregulated the expression of AR in a dose- and time-dependent fashion. Zinc-mediated AR suppression accordingly inhibited the androgen-mediated transactivation and expression of the androgen target, prostate specific antigen (PSA). This phenomenon resulted from facilitated protein degradation, not transcriptional control. In studies using mice bearing TRAMP-C2 subcutaneous tumors, the intraperitoneal injection of zinc significantly reduced tumor size. Analyses of both xenograft tumors and normal prostates showed reduced expression of AR and increased cell death. Considering the significant loss of intracellular zinc and the dominant growth-modulating role of AR during PCa development, loss of zinc may be a critical step in the transformation of normal cells to cancer cells. This study provides the underlying mechanism by which zinc functions as a PCa suppressor, and forms the foundation for developing zinc-mediated therapeutics for PCa.

## 1. Introduction

Prostate cancer (PCa) is the second most common cause of death by cancer in American men [1]. One of the primary treatments for PCa is hormone ablation; however, this therapy eventually fails due to adaption to the low androgen environment of PCa [2]. Both androgen and the androgen receptor (AR) play a pivotal role in the growth of normal prostates and the development and progression of PCa [3,4]. As a member of the steroid receptor superfamily, the AR is a nuclear receptor that functions as a ligand-dependent transcription factor [5]. In the absence of ligand binding, the receptor is inactivated in the cytoplasm by chaperones, such as heat shock proteins (HSPs) [6,7]. Upon binding to a ligand, such as testosterone, the AR is released from HSPs, dimerized, and rapidly translocated to the nucleus [8]. The translocated AR binds to the androgen response elements (AREs) on target promoters, where it interacts with other factors and activates target gene transcription [9]. The most widely studied and clinically used AR target gene is prostate specific antigen (PSA) [10]. PSA is a chymotrypsin-like serine protease, and its expression is tightly regulated by the AR [11].

Zinc is a metal ion that is required for the activity of many enzymes and transcription factors [12]; thus, it is essential for the optimal growth and development of the human body [13,14]. Early studies revealed that the human body contains 2–4 g of zinc, with the highest content located in the prostate [15]. In 1967, Ferenc et al. reported that the zinc content in prostate carcinomas was much lower than that in normal prostatic epithelial cells [16]. Zinc concentration was highest in benign prostate hyperplasia compared with normal prostates, while the lowest concentrations were observed in malignant prostate carcinomas [17,18]. The loss of zinc during prostate tumorigenesis is not yet clearly understood. Many researchers have demonstrated the effects of zinc on prostate cells and the regulation of zinc in normal prostates and malignant PCa. The re-introduction of physiological levels of zinc was found to inhibit >50% of androgen-responsive cell growth [19]. Subcutaneously implanted minipumps with zinc chloride (ZnCl_2_) over a 5-week period resulted in a significant decrease in PC3 tumor growth in tumor-bearing animals [20]. Similar tumor suppressor effects of zinc have also been shown in other types of tumors. For example, zinc depletion promoted esophageal tumorigenesis, which was associated with increasing tumor size and cancer stage [21]. Zinc also inhibited the proliferation of colon cancer cells [22]. Therefore, zinc appears to negatively affect tumor cell growth. However, the mechanism underlying this action is not clear. The most studied zinc target is the nuclear factor kappa-light-chain-enhancer of activated B cells (NF-κB) signaling pathway [23,24,25]. Zinc inhibits the activation of NF-κB in PCa cells, likely by blocking IκB kinase (IKK) to reduce the invasive potential of PCa cells. In this study, we attempted to determine the role of zinc in terms of PCa growth modulation. We demonstrated that growth inhibition due to zinc specifically affects AR-retaining (AR(+)) PCa cells, since zinc post-transcriptionally targets AR expression. These results are supported by in vivo studies and may provide valuable insight for the future use of zinc for PCa treatment.

## 2. Results

### 2.1. Zinc Inhibits the Proliferation of AR(+) PCa Cells

**Figure 1:**
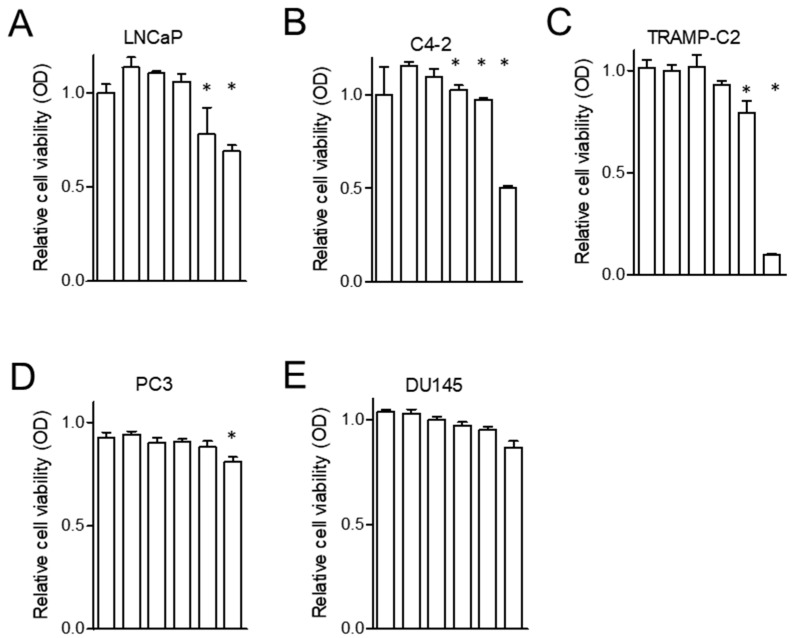
Zinc inhibits the proliferation of PCa cells. PCa cell lines were treated with increasing doses of zinc. After 48–72 h, cells were stained with MTT reagent and the absorbance at 570 nm was measured. (**A**) LNCaP treated with 0, 10, 50, 100, 150, and 200 µM zinc for 48 h. (**B**) C4-2 treated with 0, 10, 50, 75, 100, and 150 µM zinc for 48 h. (**C**) TRAMP-C2 treated with 0, 10, 20, 40, 50, and 100 µM zinc for 48 h. (**D**) PC3 treated with 0, 10, 20, 50, 75, 100 µM zinc for 72 h. (**E**) DU145 treated with 0, 50, 100, 150, 200, and 300 µM zinc for up to 72 h. * *p* < 0.05.

To demonstrate the effect of zinc on PCa cell proliferation, zinc in the form of ZnCl_2_ was employed as previously described [20]. The AR(+) PCa cells used were LNCaP, C4-2 and TRAMP-C2, while the AR-deficient (AR(−)) PCa cells used were PC3 and DU145. Each cell line was treated with ZnCl_2_ at concentrations of 0–300 µM. Cell growth was measured by 3-(4,5-dimethylthiazol-2-yl)-2,5-diphenyltetrazolium bromide (MTT )assay after 48–72 h incubation with zinc salt. As shown in Figure 1, the cell growth of all of the AR(+) PCa cells was inhibited by zinc, while all of the AR(−) PCa cells were minimally affected by zinc (by Tukey’s HSD analysis, *p* < 0.001). The proliferation of the LNCaP and C4-2 cells was inhibited after the cells were incubated with 200 µM zinc for 48 h (*p* < 0.001 by 2-way ANOVA for each cells). The TRAMP-C2 PCa cells were more sensitive to zinc-induced cytotoxicity (*p* < 0.001), as indicated by the reduction of their growth to 10% with 100 µM zinc treatment. However, the AR(−) PC3 and DU145 cells were minimally affected by zinc concentrations of up to 300 µM for 72 h. This result suggests that ZnCl_2_ functioned as a negative growth regulator for the AR(+) PCa cells.

### 2.2. Zinc Suppresses the Expression of AR and PSA, and AR-Mediated Transactivation in AR(+) PCa Cells

**Figure 2:**
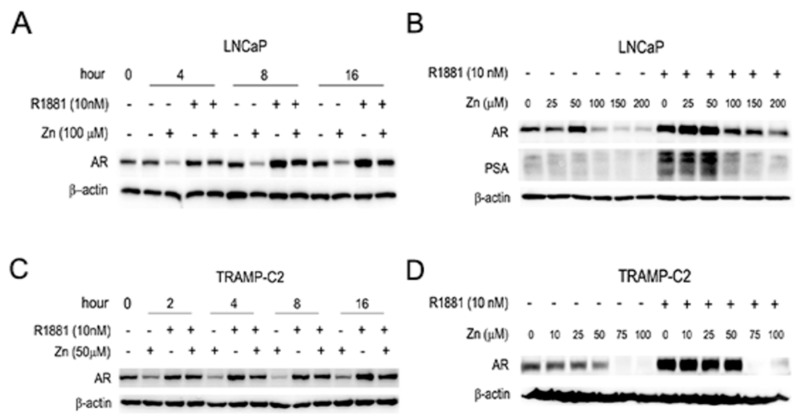
Zinc reduces AR and PSA protein expression. Western blot analysis showed the inhibitory activity of zinc on the expression of AR and PSA in human LNCaP (**A**,**C**) and mouse TRAMP C2 (**B**,**D**) PCa cells. LNCaP and TRAMP-C2 cells were grown in androgen-deprived conditions in the absence or presence of R1881.

Androgen plays a central role in the proliferation of PCa and regulates various androgen-target genes, such as PSA. To elucidate the mechanism by which zinc inhibits the proliferation of AR(+) PCa cells, human LNCaP and mouse transgenic adenocarcinoma of the mouse prostate (TRAMP)-C2 PCa cells were chosen for this experiment. Using Western blot analysis, zinc was shown to suppress the expression of AR in both the presence and absence of synthetic androgen, R1881, in both LNCaP and TRAMP-C2 cells (Figure 2). 

**Figure 3:**
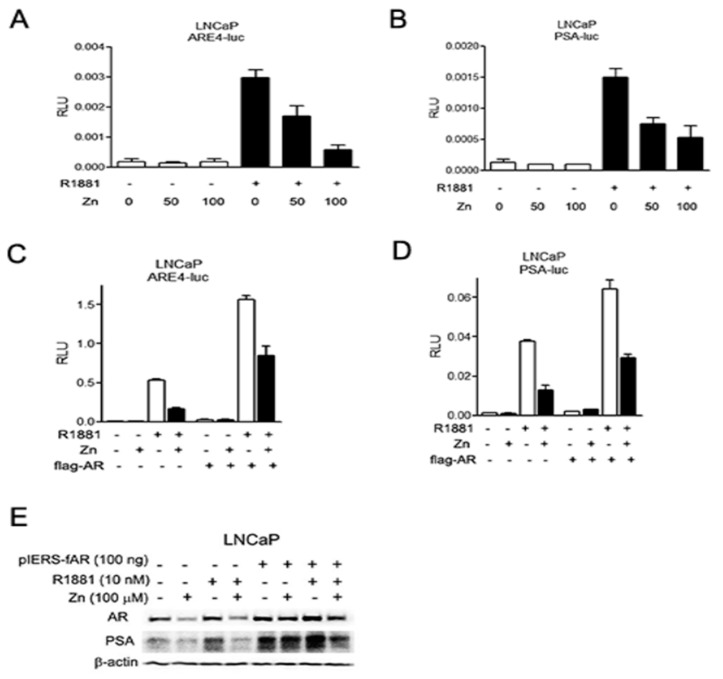
Zinc inhibits androgen-stimulated AR transcriptional activity in AR(+) PCa cells. Cells were treated with either zinc or synthetic androgen, R1881, and luciferase assays were performed 24 h post-transfection. (**A**,**B**) The effect of zinc on ARE4-luc (**A**) and p61-luc (**B**) was tested in LNCaP cells in the absence and presence of R1881. (**C**,**D**) Forced expression of AR in the presence of zinc partly restored androgen-stimulated AR activity. (**E**) The effect of exogenous AR on PSA expression was demonstrated by Western blot analysis.

It has been reported that androgen alone can stimulate the expression of AR [26]. While zinc significantly suppressed AR expression in androgen-deprived conditions, zinc also downregulated androgen-stimulated AR expression (Figure 2A,C). Consequently, the zinc-mediated downregulation of AR proteins reduced PSA expression (Figure 2B). The suppression of AR expression by zinc was accomplished in a time- and dose-dependent manner. The effect of zinc on AR expression occurred early, at 2–4 h after zinc treatment in both LNCaP and TRAMP-C2 cells, regardless of the presence of R1881 (Figure 2A,C). AR suppression was maximal at 200 µM zinc in LNCaP cells, whereas it was maximal at 75 µM in TRAMP-C2 cells. This result concurs with those of the proliferation assay (Figure 1), indicating that the TRAMP-C2 cells were more sensitive to zinc-mediated growth suppression. We further studied the effect of zinc on promoters containing AREs using reporter transcription analysis. We employed a reporter system using either an artificial promoter containing four copies of ARE (ARE4-luc) or the entire PSA promoter (p61-luc), as previously described [27]. Zinc significantly suppressed the transactivation activity of ARE (by lineal regression *r*^2^ = 0.950, *p* = 0.003; Figure 3A) and PSA in LNCaP cells (by lineal regression *r*^2^ = 0.833, *p* = 0.001; Figure 3B), especially in the presence of androgen. Since we hypothesized that the zinc-mediated suppression of AR transactivation was accomplished through down-expression of AR, we induced AR expression in LNCaP cells. Exogenous FLAG-tagged AR partly restored ARE activity in the presence of androgen (by Student’s *t*-test *p* = 0.009; Figure 3C), whereas it did not alter PSA activity (by Student’s *t*-test *p* = 0.707; Figure 3D). Western blot analysis, however, confirmed that zinc-mediated AR suppression and consequential PSA downregulation was overcome by the addition of AR (Figure 3E). These results suggest that zinc markedly inhibits androgen-stimulated AR activity and its target gene expression by downregulation of AR expression.

### 2.3. Zinc-Mediated AR Downregulation is Mediated by Facilitating Proteasomal Degradation

**Figure 4:**
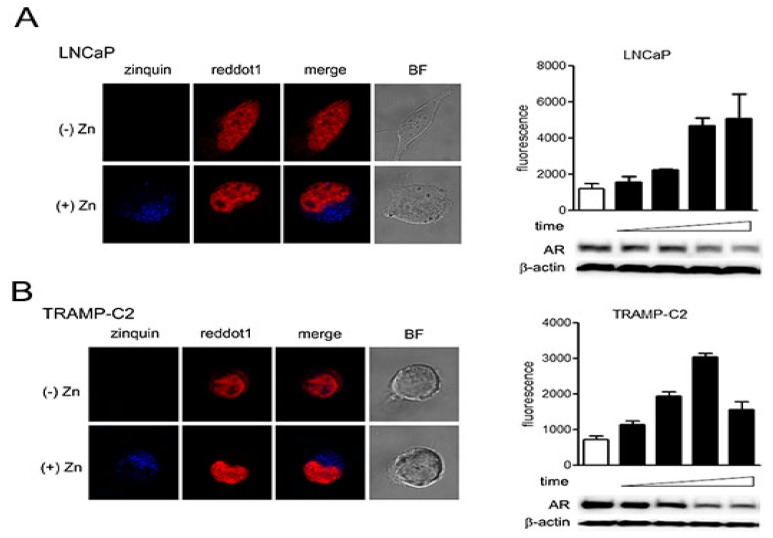
Exogenous zinc is primarily localized to the cytoplasm. (**A**,**B**) (left panels) Cells were seeded in 4-well chambers to reach 60% confluence, then treated with zinc at 150 µM (LNCaP) or 75 µM (TRAMP-C2) for 6 h. Cells were then treated with 20 µM zinquin and incubated for 30 min at 37 °C. Cells were washed and treated with RedDot1 and observed by confocal microscopy. (**A**,**B**) (right panels) Fluorescence intensities were measured at 0, 1, 2, 4, and 6 h and are expressed as mean ± SD. Cells monitored by confocal microscopy were also used for protein analysis.

The loss of zinc in PCa cells is believed to be partly due to genetic alterations in the expression of zinc import transporters such as Zip1, which has suppressed expression in PCa [28,29]. Zinc can exist in the cytosol in free form, in protein-bound form, and as vesicular zinc [30]. To determine if our approach to force exogenous zinc into the cells was feasible, we performed a zinc tracking assay using zinquin ethyl ester as a fluorescent probe to localize zinc, as previously described [31]. After treating LNCaP and TRAMP-C2 cells with zinc, we added zinquin to localize exogenous zinc by confocal microscopy. Compared to the untreated cells, zinc was localized to the cytoplasm of both types of cells (Figure 4A,B, left panels). The zinc-treated cells were also collected to determine AR expression by Western blot analysis (Figure 4A,B, right panels). As zinc concentration was gradually increased, AR expression was downregulated (by lineal regression analysis *p* < 0.001, *p* < 0.01, respectively). These results suggest that exogenous zinc effectively entered the LNCaP and TRAMP-C2 PCa cells and localized in the cytosol but not in the nucleus, which resulted in the effective suppression of AR expression.

**Figure 5:**
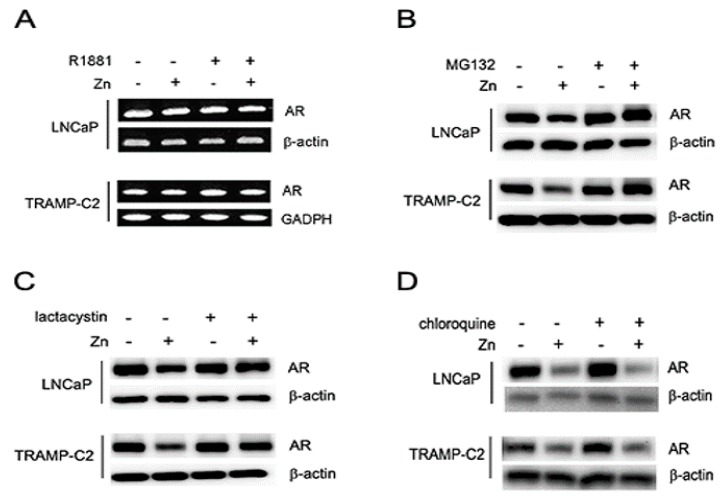
Zinc-mediated suppression of AR expression is facilitated by proteasomal degradation. (**A**) AR RNA levels were verified by RT-PCR analysis. (**B**,**D**) LNCaP (150 µM) and TRAMP-C2 (75 µM) cells were treated with zinc for 4 h followed by the addition of either MG132 (25 µM, (**B**), lactacystin (1 µM), (**C**) or chloroquine (10 µM). (**D**) Cells were analyzed by Western blotting.

To investigate how zinc suppresses AR expression, we first performed reverse transcription polymerase chain reaction (RT-PCR) using primers designed to recognize both human and mouse AR cDNA, showing that zinc did not alter the transcription levels of AR in both LNCaP and TRAMP-C2 cells (Figure 5A). To verify that zinc-mediated AR suppression was accomplished through protein degradation, we treated the cells with several commonly used protein inhibitors. Both proteasome inhibitors MG132 and lactacystin protected the degradation of AR in LNCaP and TRAMP-C2 cells (Figure 5B,C). In contrast, the lysosome inhibitor chloroquine did not protect AR degradation by zinc in either cell line (Figure 5D). Therefore, the suppressed expression of AR by zinc occurred at the post-transcriptional level and was regulated by the ubiquitin-proteasome system and not the lysosomal degradation system.

### 2.4. Zinc Inhibits PCa Growth In Vivo

**Figure 6:**
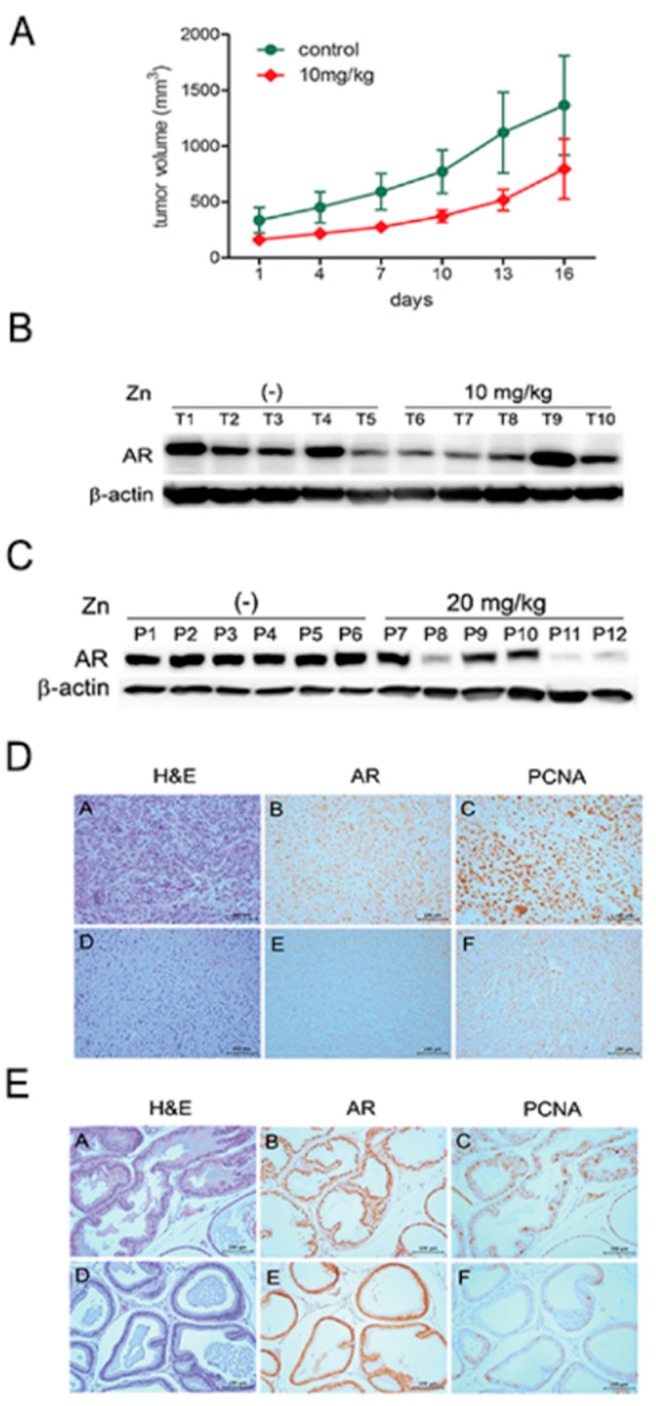
Zinc suppresses PCa cell growth in vivo. (**A**) Mouse TRAMP-C2 cells were subcutaneously inoculated into syngeneic mice and xenograft tumor growth was monitored after intraperitoneal injection of 10 mg/kg ZnCl_2_ twice a week. (**B**) After animals were sacrificed, tumors were collected and lysed, and AR expression was detected by Western blotting. (**C**) Seven-week-old male mice were treated with 10 or 20 mg/kg ZnCl_2_ twice by peritoneal injection. Nine days later, whole prostates were isolated and subjected to Western blot analysis. (**D**,**E**) Immunohistochemical analyses of tumors from (**A**,**D**) and normal prostates from (**C**,**E**) were performed. Bars indicate 100 µm.

The growth of the AR(+) PCa cells was determined to be inhibited by ZnCl_2_ by in an in vitro proliferation assay. Zinc effectively reduced the number of viable cells in a dose-dependent manner in human LNCaP and C4-2 cells and mouse TRAMP-C2 cells. We pursued zinc-mediated growth suppression in vivo using syngeneic mice accommodating TRAMP-C2 cells. It is known that the subcutaneous administration of TRAMP-C2 cells forms tumors with a histology similar to that of advanced tumors in TRAMP mice [32]. Seven-week-old male C57BL/6J mice were subcutaneously injected with TRAMP-C2 cells. When tumor volumes reached ~150 mm^3^, ZnCl_2_ was injected into the mice by intraperitoneal administration twice a week. Since similar studies using the same chemical formulation of ZnCl_2_ dosed mice at 28 mg/kg body weight [33], we chose to use either 10 mg/kg or 20 mg/kg. The growth of the implanted tumors was monitored every two days for up to 16 days after zinc treatment. Although 20 mg/kg ZnCl_2_ was lethal to several mice, the 10 mg/kg-injected group showed growth suppression of TRAMP-C2 tumors compared to the PBS-treated group. There were statistically significant differences between groups and days (by repeated measures ANOVA *p* < 0.00; Figure 6A). Animals were sacrificed at three weeks after zinc inoculation to harvest the TRAMP-C2 tumors. The effect of zinc on normal prostates was also observed. Seven-week-old male mice were treated with PBS, 10 mg/kg ZnCl_2_, or 20 mg/kg ZnCl_2_. In addition to the prostate, major organs, including the liver, lungs, heart, kidney, spleen, and intestines, were harvested. Western blot analysis revealed a significant decrease in AR protein expression in 10 mg/kg zinc-treated tumors (Figure 6B).

Mice treated with 20 mg/kg zinc showed a marked decrease in prostate AR expression, while 10 mg/kg zinc-treated mice did not show any alteration (Figure 6C). Tissues were fixed with formalin, embedded in paraffin, and subjected to histological and immunohistochemical analyses. The expression levels of both the AR and the proliferating cell nuclear antigen (PCNA), and the proliferation index were diminished in the zinc-treated tumors (Figure 6D) and prostates (Figure 6E). The major organs did not show any morphological changes as determined by routine histological microscopic examination. These results suggest that zinc suppressed not only the growth of tumor xenografts, but also the development of the normal prostate.

## 3. Discussion

Many studies have focused on the AR to determine the progression of PCa after hormone ablation therapy, since the AR plays an important role in the survival of this devastating disease. In this study, we demonstrated that ZnCl_2_ downregulated AR protein expression in AR(+) PCa cells. Zinc-mediated AR suppression was observed in both the presence and absence of androgen; however, the suppression of AR was more prominent in androgen-rich conditions. Moreover, zinc suppressed AR-mediated transcriptional activity and expression of its target protein by downregulation of AR proteins. Subsequently, zinc inhibited the proliferation of AR(+) PCa cells, including LNCaP, C4-2 and TRAMP-C2, whereas zinc did not affect the proliferation of AR(−) PCa cells, including PC3 and DU145. Further in vivo studies using tumor-bearing mice showed that intraperitoneal injection of ZnCl_2_ also suppressed the growth of AR(+) TRAMP-C2 cells in which AR expression was suppressed. Considering that normal prostates contain high concentrations of zinc, and loss of zinc occurs during prostate carcinogenesis, the zinc-mediated blockage of androgen signaling by the downregulation of AR expression would appear to contribute to PCa cell proliferation; low levels of zinc during prostate carcinogenesis would increase AR expression and favor cell proliferation, and could therefore be one of the critical steps in transformation. We also observed that high doses of zinc affected AR expression and the proliferation of normal epithelial cells in the prostate, suggesting that a balance of zinc is required to maintain a healthy prostate.

Low doses of zinc may not reach the biological threshold and, at higher doses, zinc may become ineffective due to zinc toxicity [34]. The total cellular zinc concentration for most mammalian cells is typically in the range of 100–500 μM [35]. In contrast, the zinc concentration of epithelial cells in the peripheral zone of the prostate is in the range of 800–1500 μM [36]. Zinc concentration in wet tissue is generally known to be over 1 mM, but only limited bioavailable free zinc is available [16,37,38,39,40,41]. The distribution of zinc in the cells is about 30–40% in the nucleus and 50% in the cytoplasm, with the remainder in the cell wall or the cell membrane [42]. Franklin et al. have divided total intracellular zinc (0.2–1 mM) into three pools, including tightly bound zinc as an immobile and unreactive pool, loosely bound zinc, and a reactive pool of free zinc ions. About 90% of cytoplasmic zinc is bound to immobile macromolecules, mostly proteins, whereas only 10% is bound to mobile low molecular weight ligands [43].

Many studies have been performed to determine the role of zinc in PCa development. Our results are consistent with previous studies that link zinc to PCa growth. Treatment with zinc sulfate between 200–600 µM induced citrate production in PC3 PCa cells, and further inhibited the growth of PC3 cells injected in nude mice [44]. The direct intra-tumor injection of zinc acetate (0.6 µM) halted PC3 tumor growth and extended the survival of the animals while causing no cytotoxicity to other tissues [45]. The intraperitoneal injection of ZnCl_2_ of up to 15 mg/kg body weight in Swiss albino mice was not toxic, but caused chromosomal aberrations in bone marrow cells and rupture of the epididymal epithelium [46,47]. Marshall et al. also reported that the intraperitoneal injection of ZnCl_2_ at three different doses (5, 10, and 30 mg/kg) in SW480 colon cancer-bearing severe combined immunodeficiency (SCID) mice promoted a zinc-mediated promoter response [34]. The treatment of mice with 30 mg/kg ZnCl_2_ was toxic; however, 10 mg/kg showed the highest activity of gastrin promoter activation. In this study, the treatment of SW480 cells up to 200 mM ZnCl_2_ effectively enhanced promoter activity. In our study, therefore, we decided to use 10–20 mg/kg ZnCl_2_ for in vivo use and up to 200 µM ZnCl_2_ for in vitro study. As expected, the intraperitoneal injection of 20 mg/kg ZnCl_2_ was detrimental to animals, while 10 mg/kg ZnCl_2_ moderately slowed TRAMP-C2 PCa cells and consequently downregulated AR expression.

While zinc is known to inhibit cancer growth, the mechanism of its action is unclear [19,20,21,45]. The best known intracellular target of zinc is the transcription factor NF-κB, which affects the development and progression of PCa by regulating the expression of genes involved in proliferation, apoptosis, angiogenesis, and tumor invasion and metastasis [48,49]. NF-κB transcription factors are composed of homo- and hetero-dimers. The most common NF-κB dimer comprises p50 and p65 subunits, and binds to IκB. Stimulation by TNF-α leads to the phosphorylation and degradation of IκB by IKK. This releases NF-κB, which is translocated to the nucleus where it induces the expression of numerous regulatory genes [50]. Overexpression of p65, or both p65 and p50, activates the AR promoter in a dose-dependent manner, whereas overexpression of one of its inhibitors, such as nuclear factor of kappa light polypeptide gene enhancer in B-cells inhibitor (IκB), inhibits AR promoter activation [51]. In addition, p65 increases endogenous AR mRNA levels 2.5-fold, and promotes PCa cell proliferation. It has also been demonstrated that ~40% of AR binding activity is lost as a result of exposure to zinc [52]. Physiological levels of zinc inhibit the activation of NF-κB in PCa cells, likely by blocking IKK [24,53]. We also observed the inhibition of NF-κB by zinc via the regulation of IKKα [54]. This evidence suggests a link between zinc and the AR signaling pathways, with a further relation to NF-κB signaling. Therefore, it is possible that zinc regulates two major signaling pathways for PCa growth and proliferation, suggesting that a balance of intracellular zinc levels may be important for the regulation of NF-κB signaling in androgen-independent PCa cells. Our study demonstrates that zinc inhibits PCa cell growth by downregulation of AR protein expression. The delivery of exogenous zinc may be an effective method to prevent PCa and treat PCa patients. Our findings provide a promising approach for the effective treatment of human PCa by way of targeting AR and possibly NF-κB in both the presence and absence of androgen.

## 4. Materials and Methods

### 4.1. Cell Culture

Human PCa cell lines LNCaP, PC3 and DU145 were purchased from American Type Culture Collection (ATCC) (Manassas, VA, USA). Human C4-2 cells were provided by Leland Chung (Cedars-Sinai, Los Angeles, CA, USA). The human cells were routinely cultured in Roswell Park Memorial Institute (RPMI) media. Mouse TRAMP-C2 cells were also purchased from ATCC and cultured in Dulbecco’s Modified Eagle Medium media. All media were supplemented with 5% fetal bovine serum (FBS), and 1% penicillin-streptomycin at 37 °C in 5% CO_2_; 95% atmosphere. ZnCl_2_ at ≥99.9% purity was purchased from Sigma-Aldrich (St. Louis, MO, USA). For this study, we decided on a concentration of ZnCl_2_ at the LD50 for each cell type in vitro, and 10–20 mg/kg for in vivo use. Up to 200 µM ZnCl_2_ was used for the in vitro proliferation assay, and 1.8 µM was used for the animal experiments. Synthetic testosterone R1881 was obtained from NEN Life Science (Boston, MA, USA) and used at a final concentration of 10 nM for all experiments.

### 4.2. Transient Transfection

Cells were seeded the day before transfection to reach at least 60% confluence for the experiments. To eliminate the effect of hormones, the cells were grown in 5% charcoal dextran-treated (CDT) FBS for three days before transfection. The cells were transfected with either ARE-4-luc or p61-luc (200 ng) and renilla vector (20 ng) with the assistance of Lipofectamine 2000 (Invitrogen, Carlsbad, CA, USA). After 6 h, cells were washed and fed with a medium containing 5% FBS. The cells were treated with ZnCl_2_, and/or R1881 as required. After 24 h, the cells were washed with PBS and lysed with 100 µL passive lysis buffer. Then, luciferase activity was assayed as relative light units (RLU) using the Dual-Luciferase Reporter Assay System (Promega, Madison, WI, USA) according to the manufacturer’s protocol. The transfection experiments were performed in triplicate and the results are reported as the mean ± SD.

### 4.3. Western Blot Analysis

LNCaP and TRAMP-C2 cells were seeded into 24-well plates to gain 50% confluence and treated with increasing doses of ZnCl_2_. Cell lysates were then collected. Tumor and prostate tissue from mice was also collected and proteins were extracted with lysis buffer. Equal amounts of lysates were loaded onto a 10% Bis-Tris gel and were separated by electrophoresis (Bio-Rad Laboratories, Hercules, CA, USA). The proteins were then transferred to a polyvinylidene difluoride (PVDF) membrane and primary antibodies were applied, followed by incubation with horse peroxidase-conjugated secondary antibodies. Anti-PSA and AR-N20 antibodies for both human and mouse AR detection were from Santa Cruz Biotechnology (Santa Cruz, CA, USA) and anti-β-actin antibodies were from Sigma-Aldrich. The blots were developed using an enhanced chemiluminescence (ECL) detection system (Thermo Fisher Scientific, Rockford, IL, USA) and imaged with an LAS4000 luminescent image analyzer (Fuji, Tokyo, Japan).

### 4.4. RNA Isolation and RT-PCR

Total RNA was extracted with TRIzol-chloroform (Invitrogen, Carlsbad, CA, USA), and reverse transcription (RT) was performed using the GoScript^TM^ reverse transcription system (Promega). The cDNA was then amplified by PCR using GoTag DNA polymerase (Promega). The sequences of the human AR primers were: 5′-GGATGAGGAACAGCAACCTTCAC-3′ and 5′-ATGGACACCGACACTGCCTTACAC-3′. The sequences of the human β-actin primers were: 5′-ACTCTTCCAGCCTTCCTTC-3′ and 5′-ATCTCCTTCTGCATCCTGTC-3′. The mouse AR primer sequences were: 5′-TATGTGCCAGCAGAAACGATTGTA-3′ and 5′-TGTGCATGCGGTACTCATTGAAAA-3′. The mouse Glyceraldehyde 3-phosphate dehydrogenase GAPDH primer sequences were: 5′-ACCACAGTCCATGCCATCAC-3′ and 5′-TCCACCACCCTGTTGCTGTA-3′.

### 4.5. In Vitro Cell Proliferation Assay

Cells (1 × 10^4^) were plated on 24-well plates, treated with ZnCl_2_ the next day, and then incubated for 2–3 days. Then, cells were stained with 50 µL of 5 mg/mL MTT solution and incubated at 37 °C for 2–4 h. The reactions were stopped by adding 500 µL of dimethyl sulfoxide DMSO solution and the absorbance was measured at 570 nm using a microplate reader with version 6 of SOFTmax PRO software (Molecular Devices, Sunnyvale, CA, USA).

### 4.6. Confocal Microscopy

Intracellular zinc levels were determined using zinquin ethyl ester (Enzo Life Sciences, Farmingdale, NY, USA), a cell permeable UV-excitable dye that fluoresces in the blue region upon binding to zinc. To confirm the localization of exogenous zinc within the PCa cells, the cells were plated in 4-well chambers and treated with ZnCl_2_ for 6 h, and 25 µM of zinquin ethyl ester was added for 30 min. The cells were washed, fixed, permeabilized, and stained with 1X RedDot^TM^1 (Biotium, Fremont, CA, USA) for nuclear staining. Fluorescence was monitored under a Zeiss LSM700 confocal microscope (Zeiss, Oberkochen, Germany) at far-red wavelength for nuclei staining and violet for zinc detection. Fluorescence intensity was also measured using a dark-bottom 96-well plate in a Tecan reader (Tecan Group Ltd., Männedorf, Switzerland).

### 4.7. Immunohistochemistry

Tumor and normal prostate tissues were collected from mice and fixed with 10% formalin, then dehydrated for paraffin-embedded sectioning. Tissue sections were deparaffinized in a dry oven for 1 h at 60 °C. Endogenous peroxidase activity was stopped by incubating slides in a solution of methanol containing 3% H_2_O_2_. Before the primary antibody was applied, nonspecific reactivity was blocked with the appropriate blocking buffer, followed by incubation with anti-AR-N20 and-pCNA antibodies (Santa Cruz Biotechnology) overnight. Avidin-biotin-peroxidase complex-conjugated (Vector Laboratories Inc., Burlingame, CA, USA) secondary IgG antibodies were applied, and the colorimetric signals were recorded using 3,3′-Diaminobenzidine substrate. Slides were counterstained by immersing in hematoxylin for microscopic evaluation.

### 4.8. In Vivo Study

Male C57BL/6J mice were used for in vivo experiments. All experiments were performed in accordance with our institution’s guidelines for animal care. The mice were housed and monitored under constant humidity and temperature. TRAMP-C2 cells (2.5 × 10^6^) were subcutaneously inoculated to mouse flanks to induce tumors. When tumor volumes reached ~150 mm^3^, the mice were randomly divided into 2 groups: The control group injected with water, and the experimental group injected with 10 mg/kg ZnCl_2_. Mice were injected intraperitoneally every 3 days and tumor size was measured for 2 weeks. Tumor volume was calculated using the following equation: length × width^2^ × 0.5. In addition, normal mice were injected intraperitoneally with 20 mg/kg ZnCl_2_ twice a week to observe the effect of zinc in normal prostates. The control mice were inoculated with water instead of ZnCl_2_. The animals were sacrificed 3 weeks after inoculation, and tumor and prostate tissue was collected for further analyses.

### 4.9. Statistical Analysis

All values are presented as mean ± SD. Data were analyzed by Student’s *t*-test (GraphPad Prism Software, La Jolla, CA, USA), one-way and two-way ANOVA, linear regression (SPSS), Tukey’s honestly significant difference (HSD) test (SPSS), and repeated measurements analysis (SPSS). *p* < 0.05 was considered significant.
